# Prevalence, Associated Factors and Predictors of Depression among Adults in the Community of Selangor, Malaysia

**DOI:** 10.1371/journal.pone.0095395

**Published:** 2014-04-22

**Authors:** Siti Fatimah Kader Maideen, Sherina Mohd. Sidik, Lekhraj Rampal, Firdaus Mukhtar

**Affiliations:** 1 Department of Psychiatry, Faculty of Medicine & Health Sciences, Universiti Putra Malaysia, Selangor, Malaysia; 2 Department of Community Health, Faculty of Medicine & Health Sciences, Universiti Putra Malaysia, Selangor, Malaysia; Institute of Psychiatry, United Kingdom

## Abstract

**Introduction:**

Depression is one of the most common mental health disorders and is an emerging public health problem. The objectives of this paper were to determine the prevalence of depression, its associated factors and the predictors of depression among adults in the community of Selangor.

**Methods:**

A cross sectional study was conducted in three districts in Selangor, from 11th June to 30th December 2012. The sampling frame was obtained from the Department of Statistics Malaysia (DOS) in May 2012, using the National Population and Housing Census 2010. Adults aged 18 years and above, living in the selected living quarters were approached to participate in the study and requested to complete a set of questionnaires.

**Results:**

A total of 1,556 out of 2,152 participants participated in this study, giving an overall study response rate of 61.90%. Patient Health Questionnaire 9 (PHQ-9) was used to determine the presence of depression. The prevalence of depression was 10.3%, based on the PHQ-9 cut off point of 10 and above. Based on multiple logistic regression analysis, the predictors of depression were presence of anxiety, serious problems at work, unhappy relationship with children, high perceived stress, domestic violence, unhappy relationship with spouse, low self-esteem, unhappy relationship with family, serious financial constraint and presence of chronic diseases. When reanalyzed after removing anxiety, high perceived stress and low self-esteem, additional predictors of depression were found to be serious marital problems and religiosity.

**Conclusion:**

The prevalence of depression in this study is similar to that found in other studies. Findings from this study are being used as baseline data to develop an effective program to assist in the management of common mental health disorders in the community, in particular depression. The identification of predictors of depression in the community is important to identify the target population for the program.

## Introduction

Depression is one of the most common mental health disorders and is represented as an emerging public health problem [1]. According to the Diagnostic and Statistics Manual of Mental Disorders V (DSM V), depression is characterized by the presence of five or more symptoms for a period of 2-week and represent a change from previous functioning, with at least one of the symptom of depressed mood or loss of interest or pleasure [2]. Other symptoms include significant weight loss when not dieting or weight gain, insomnia or hypersomnia, psychomotor agitation or retardation, fatigue, feelings of worthlessness or guilt, inability to concentrate and recurrent thoughts of death or suicide.

Depression is also a major cause of morbidity and disability. Its burden of disease ranks high in many countries [3]. Depression is ranked as the fourth disorder in the global burden of disease and by the year 2030, it is expected to be the highest disorder in high-income countries [1]. It attributes about 12% of total Years Lived with Disability (YLD) in terms of disease burden [4].

In England, results from the Adult Psychiatric Morbidity Survey showed that 17.6% of the adult population met diagnostic criteria for at least one common mental disorder, among which 2.6% of them had depressive episode [5]. The National Survey on Drug Use and Health (NSDUH) in United States among adults aged 18 years and above in 2010 reported that 6.8% of adults in the population had one major depressive episode (MDE) in the previous year, accounting for 15.5 million people [6]. The prevalence of MDE was higher among females, those with age group of 18–25 years and those who were unemployed.

Based on the data from the Behavioral Risk Factor Surveillance System (BRFSS) from 2006 and 2008, the Centres for Disease Control and Prevention (CDC) of the United States of America reported that 9.1% of the adults had current depression, including 4.1% who met the criteria for major depression [7]. The 12-months and lifetime prevalence of DSM-IV Major Depressive Disorder (MDD) in United States (US) which was conducted from year 2001 to 2002 was 5.28% and 13.23% respectively [8].

The 2007 National Survey of Mental Health and Wellbeing in Australia showed that 45% of the population aged 16–85 years had lifetime prevalence of any mental disorders [9]. The survey found that 6.2% of Australians had an affective disorder, among which 4.1% of them had depressive episode in the past 12-months using the World Mental Health- Composite International Diagnostic Interview (WMH-CIDI).

A population survey among subjects aged 15 years and above in Iran, involving 35 014 individuals found that 21% of the population had mental disorders, which were high among females, married, widowed and unemployed [10]. Twenty-one-percent of the population was found to have depressive symptoms. A systematic review on risk factors of depressive disorders in Pakistan by Mirza and Jenkins from 1991–2001 showed that females, low education level, and chronic difficulties in housing, finance and health were significantly associated with depressive disorders [11].

In China, face-to-face household interviews conducted among adults from November 2001 to February 2002 found lifetime and one-year prevalence of DSM-IV/CIDI major depressive episodes of 3.6% and 1.8%, respectively [12]. A household survey carried out among 6616 adults in Singapore from December 2009 to December 2010 found a 12-month and lifetime prevalence of MDD of 5.8% and 2.2% [13], whereas lifetime prevalence of mental disorders was 12.0% [14].

In Malaysia, national surveys that are being carried out every 10 years, found that mental health problems had increased from 10.7% in 1996 [15] to 11.2% in 2006 [16]. These national surveys were conducted in community households among participants aged 16 years and above by trained medical and health care professionals. The most recent national survey, National Health Morbidity Survey IV (NHMS IV) in 2011 showed that the prevalence of lifetime depression was 2.4% and current depression was only 1.8% [17]. The variation in the prevalence rates was mainly due to the different instruments used. The two initial surveys used the General Health Questionnaire (GHQ) to screen for psychiatric morbidity [15], [16] which yielded higher prevalences of psychiatric morbidity compared to the recent survey which used the Mini International Neuropsychiatry Interview (MINI), a diagnostic instrument to diagnose depression [17]. This recent survey found that current and lifetime depression were high in urban areas, females, Indians, widowed, singles, divorced and those with lower education [17].

Mental health problems, namely depression are diseases that should not be overlooked due to the great morbidity and burden. Depression causes impairment in functional well-being and decrease in quality of life [18]–[20], decrement in health [21], [22], physical distress and health problems [22]. Depression can cause impairment in a person's role at home, work, relationships and social network [13]. These can result in limitation of daily activities [22], job insecurities [23] and increased risk of early mortality due to physical disorders and suicide [24]. Therefore, there is a need to identify subpopulations that are at high risk of suffering from depression.

Early detection is crucial to reduce the consequences and sufferings due to these problems. Therefore, this study aims to determine the prevalence of depression, its associated factors and the predictors of depression among adults in the community of Selangor, Malaysia. By identifying the predictors, targeted and focused intervention could be organized to support these high risk groups in future community mental health programs.

## Methods

### Study design

A cross sectional study was conducted in three districts of the state of Selangor in Malaysia, to determine the prevalence of depression and its associated factors among adults in the community.

### Settings

**Figure 1:**
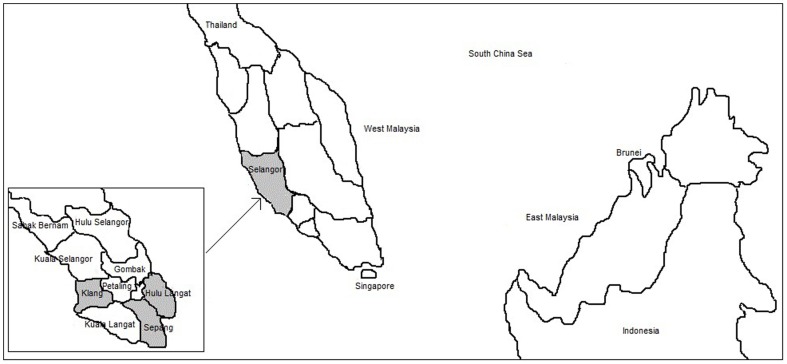
The map of Malaysia. The above figure shows the map of Malaysia. The area highlighted in grey is the state of Selangor. The enlarged view of the nine districts in Selangor is also shown. The three districts where the study was done are also highlighted in grey.

Malaysia is located in South East Asia and is made up of thirteen states and three federal territories. It is separated into two regions, Peninsular Malaysia (West Malaysia) and East Malaysia by the South China Sea. Selangor located in Peninsular Malaysia, is one of the thirteen states in Malaysia. It consists of nine districts (refer Figure 1 for the map of Malaysia).

This study was conducted in three districts of Selangor: Hulu Langat, Sepang and Klang from 11^th^ June to 30^th^ December 2012, for a duration of 29 weeks. Selangor was chosen as the study location because it is the most populated state in Malaysia, comprising of 5.46 million people, besides constituting a high level of urbanization (91.4%). Mental health problems were found to be more prevalent in urban areas compared to rural areas [16], [17].

### Sampling frame

**Figure 2:**
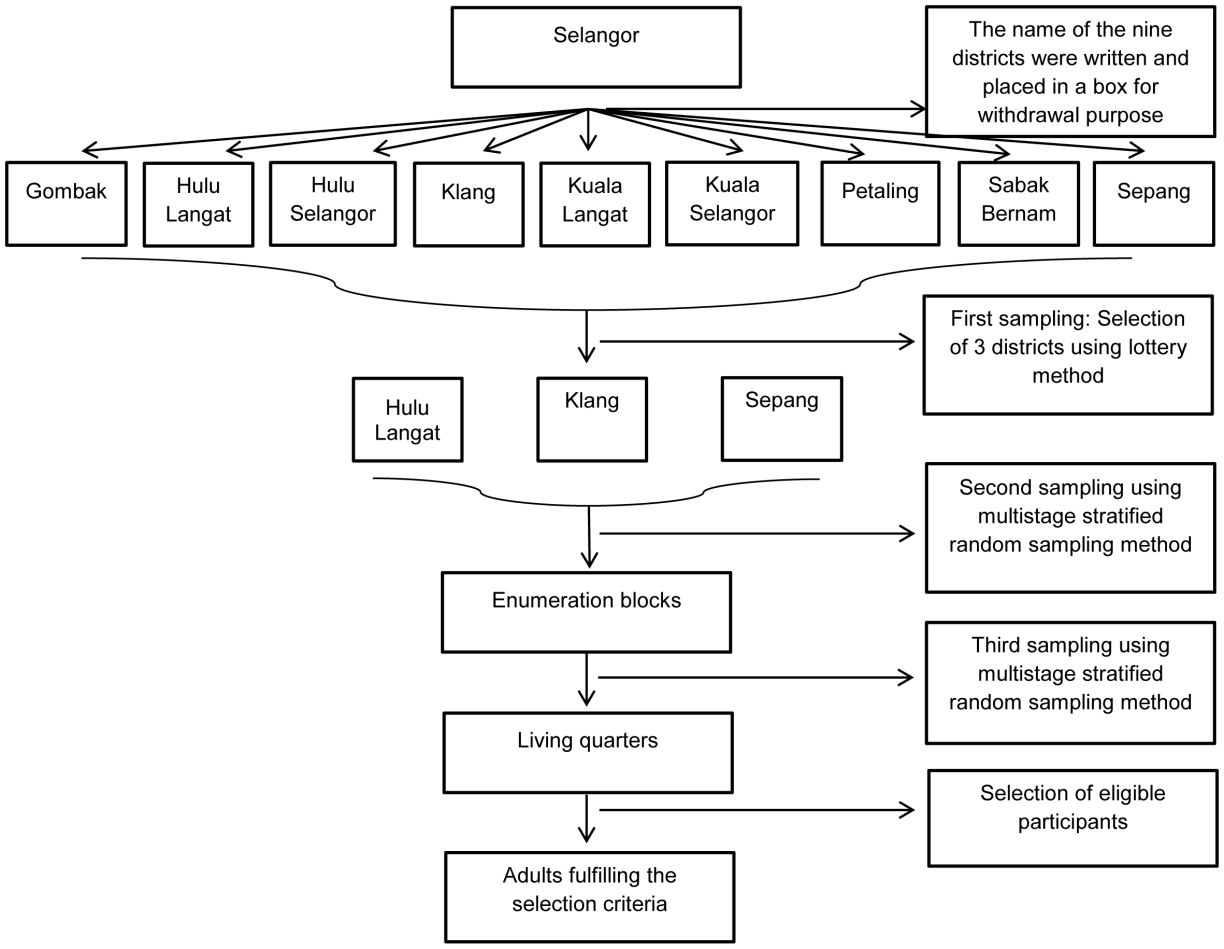
Flow chart of sampling method. The above figure shows the sampling method of this research. The sampling frame for this study was obtained from the Department of Statistics (DOS), Malaysia. A multistage stratified random sampling method was used for the selection of enumeration blocks and living quarters in each of the three districts in Selangor.

The sampling frame for this study was obtained from the Department of Statistics, Malaysia (DOS) in May 2012, using the National Population and Housing Census 2010. Maps and Enumeration Blocks (EBs) of the households in Hulu Langat, Sepang and Klang were purchased for the data collection process. A multistage stratified random sampling method with proportionate allocation was used for the selection of EBs and living quarters (LQs). LQs in this study represent households in the community. Each EB consisted of eight LQs. A total of 157 EBs were selected by the DOS. The flow chart of the sampling frame is shown as in Figure 2.

### Procedures of data collection

**Figure 3:**
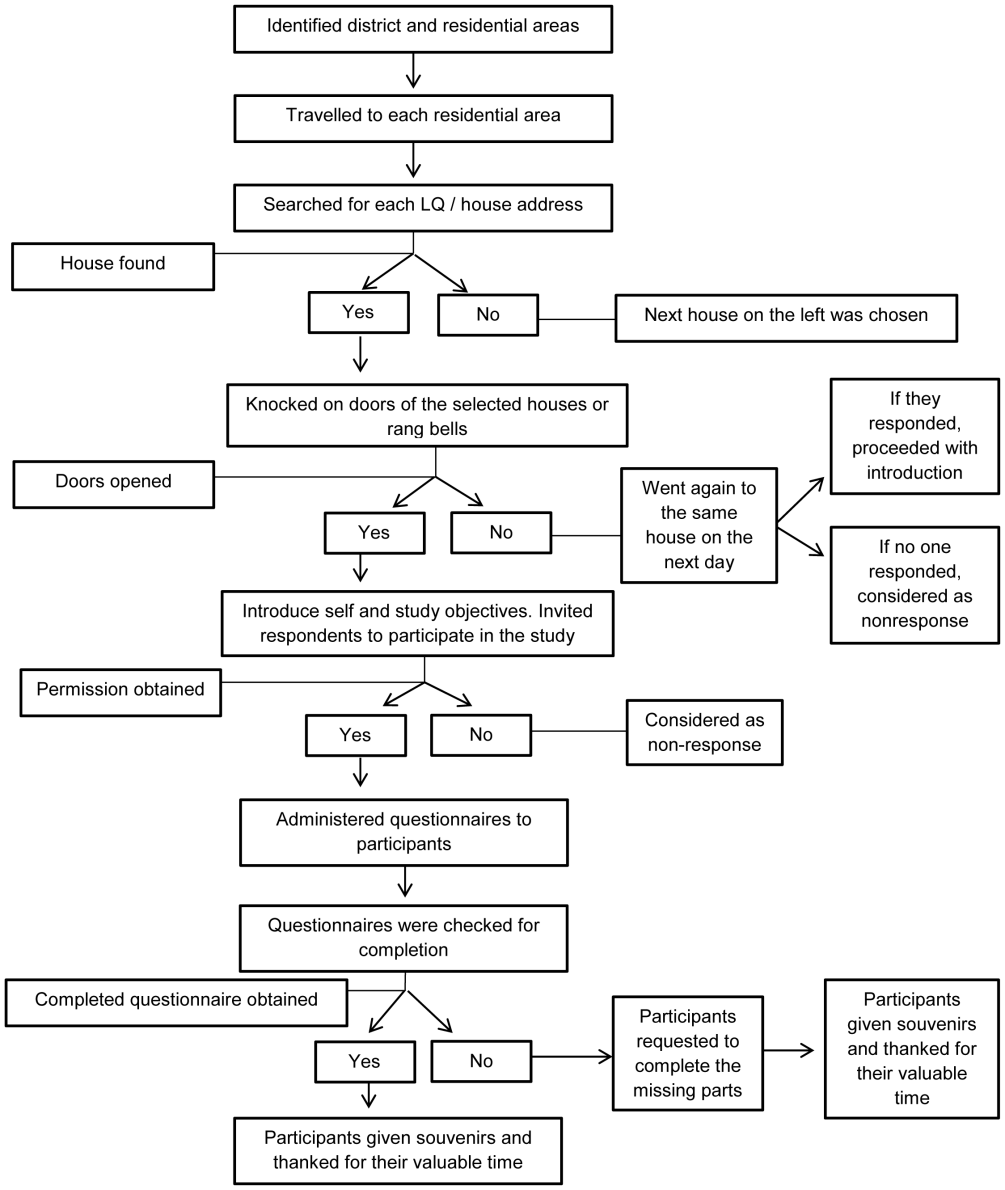
Flow chart of data collection process. The above figure shows the flow chart of the detailed data collection process, starting from the arrival at the selected houses until the end of the data collection.

Data were collected by a group of trained research assistants (RAs). The participants were briefed on the purpose of the study and were provided with the information sheet with details about the study. The questionnaires were administered upon verbal consent from the participants. Each questionnaire was checked for completeness before leaving the participant's house. The flow chart of the data collection is shown in Figure 3.

### Eligibility criteria

Adults aged 18 years old and above who were Malaysian citizens and staying in the selected LQs at the time of data collection were invited to participate in the study. Those with severe communication problems, such as total inability to comprehend both Malay and English languages were excluded from the study.

### Study size

The sample size of this study was calculated based on the estimated prevalence of poor mental health status in Malaysia, level of confidence and the margin of error desired. To calculate the sample size, the prevalence of poor mental health status of 11.2% based on the NHMS III [16], a precision of 2%, and an estimate of 50% non-response rate were taken into consideration. The final sample size of 2512 subjects was calculated.

### Instruments

The questionnaire used in this study included questions on socio-demographic characteristics, depression, presence of chronic diseases and history of mental health disorders, anxiety, stressful life events, perceived stress, domestic violence, self-esteem and religiosity.

#### Socio-demographic characteristics

Items on the socio-demographic characteristics included age, gender, ethnicity, religion, marital status, education level, employment status and income.

#### Patient Health Questionnaire-9 (PHQ-9)

The PHQ-9 was used in this study to determine the presence of depression. The Patient Health Questionnaire (PHQ) is a version of the PRIME-MD diagnostic instrument for common mental health disorders. The PHQ-9 is a self-report instrument used to determine the presence of depression based on the DSM-IV criteria. It consists of 9 items, with each item scored from 0–3, with an overall range score of 0–27. The presence of depression in this study was determined by using the cut-off point of 10 and above on the PHQ-9. A cut off point of 10 and above was used as it has the optimum level of sensitivity and specificity [25], [26]. The PHQ-9 was first validated by Kroenke, K et al among patients in primary care, and obstetrics and gynecology clinics [25]. The validated Malay version of PHQ-9 which was found to have good sensitivity and specificity was used in this study [26].

#### Presence of Chronic Diseases

The presence of chronic diseases was self-reported by the participants based on the diagnosis by medical professionals. Some of the items included in the questionnaire were ‘Have you been diagnosed with any chronic diseases’, ‘Have you been diagnosed with heart disease’ and questions on other chronic diseases, such as diabetes, stroke, hypertension and arthritis.

#### Generalized anxiety disorder-7 (GAD-7) Questionnaire

The GAD-7 is a self-report instrument used to measure anxiety and its severity, based on the criteria from DSM-IV. It consists of 7 items, with each item scored from 0–3, with an overall range score of 0–21. The presence of anxiety was determined by using the cut-off point of 8 and above on the GAD-7. A cut-off point of 8 and above was used as it has optimum level of sentivity and specificity [27]. The GAD-7 was developed and validated by Spitzer, RL et al among patients in primary care clinics [28]. The validated Malay version of GAD-7 which was found to have good sensitivity and specificity was used in this study [27].

#### Kendler's Stressful Life Events

A list of seventeen items of stressful life events by Kendler et al was used to assess stressful life events experienced by the participants [29]. Some of the items in the questionnaire are ‘Have you ever been assaulted’ and ‘Have you ever been seriously ill’. ‘A ‘yes’ to any of these 17 items was considered as positive for a particular stressful life event.

#### Cohen's Perceived Stress Scale (PSS)

The Perceived Stress Scale measures the degree to which situation in one's life is appraised as stressful [30]. It was developed by Cohen et al and was validated among college students and adults attending a smoking-cessation program. The PSS-10 was chosen over the PSS-14 as the study instrument as it had a better factor structure and internal reliability [31]. The Malay translated version of PSS-10 was used in this study. The translation was similar to the validated PSS-10 scale among private university students in Malaysia [32]. The PSS-10 is made up of 10 positive and negative worded items, which are rated on a five-point Likert scale (never, almost never, sometimes, fairly often and very often). The scale measures perceived stress in the past month. The total scores of perceived stress ranged from 0–40. Higher scores denote higher perceived stress. Since there is no specific cut-off point, the mean score of the total items was used as the cut-off point to categorize low and high perceived stress.

#### HARK questionnaire on Domestic Violence

The Malay translated version of the HARK questionnaire was used to determine domestic violence. The HARK questionnaire was developed and validated by Sohal et al among women involved in intimate partner relationship, attending general practice clinics [33]. The word HARK denotes humiliation, afraid, rape and kick. The HARK questionnaire consists of four items, which includes questions on emotional, psychological, sexual and physical abuse. The instrument assessed intimate partner violence in the past year. A ‘yes’ to any of these items was considered as presence of domestic violence. In this study, domestic violence is defined as the commission of abusive act/acts by spouse or former spouse in the last year. Due to the cultural sensitive issues and the definition of domestic violence by the Malaysian Law [34], the HARK instrument in this study was only answered by participants who were ever married.

#### Rosenberg's Self-esteem Scale (RSES)

The Rosenberg's Self-esteem Scale is a measure of global self-esteem developed by Morris Rosenberg [35]. The validated Malay version of the Rosenberg self-esteem scale was used in this study [36]. It consists of 10 positive and negatively worded items, which are rated on a four-point scale (strongly agree, agree, disagree and strongly disagree). The total scores of self-esteem ranged from 10–40. Higher scores indicate higher self-esteem. The mean score of the total items was used as the cut-off point to categorize low and high self-esteem [37].

#### DUREL questionnaire on Religiosity

The Duke University Religion Index (DUREL) was developed and validated by Koenig, HG and Bussing, A [38]. The validated Malay version of the DUREL was used in this study [39]. It is a five-item measure of religious involvement that assesses three major dimensions of religiosity: organizational religious activity (ORA), non-organization religious activity (NORA) and intrinsic religiosity (IR).

### Ethics Statement

Research ethics approval from the Medical Research Ethics Committee of the Faculty of Medicine and Health Sciences, University Putra Malaysia was obtained prior to data collection. All participants provided verbal consent, however some refused to give written consent as they were afraid that their signatures would be misused for other purposes. When the participants verbally consented to participate in the study, the research assistants ticked the consent given box in the consent form. Therefore there was documented evidence/record that the participants gave consent. The ethics committee approved of this consent procedure if the participants refused written consent.

### Statistical Analysis

Data were entered and analyzed using the IBM SPSS Statistics Version 21.0. Data were checked and verified. There were still missing data in the questionnaires, even though checking was done during data collection, however the percentage of missing data was minimal. As the part of missing data varied, data were analyzed based on the complete information for that particular instrument.

Age was expressed in mean ± standard deviation (SD). Chi square test was used to determine the association between the independent categorical variable and depression. For frequency of less than 20% in the 2×2 cell, Fisher's exact test was used instead of the Chi square test. T-test was used to compare means between groups (depressed versus non-depressed). Significant variables (p<0.25) from the Chi square, Fisher's exact test and t-test were inserted into the logistic regression model. Multivariate logistic regression analysis was employed to determine the predictors of depression after controlling for the confounders. The enter method was used. The predictors for depression was based on the p-value of <0.05.

## Results

### Participants

A total of 1556 out of 2512 participants participated in this study, giving an overall response rate of 61.90%. The reasons of non-participation in this study were time restriction due to busy and tight schedules, not feeling well and inappropriate time of survey.

Out of 1556 participants, 1545 of them (99.3%) reported their age, which ranged from 18 to 87 years, with a mean of 35.36±13.77 years. The majority of the participants was females (62.1%), Malays (66.3%), married (60.3%), and employed (53.2%).

Out of the 1556 participants, 1460 completed the PHQ-9 questionnaire (response rate 93.8%). Based on the PHQ-9 cut off point of ≥10, 150 participants had depression, giving a prevalence rate of 10.3% (95% CI: 8.74–11.86).

### Factors associated with depression

#### Socio-demographic characteristics

Among the socio-demographic characteristics, only ethnicity and marital status were found to be associated with depression (p<0.05). The prevalence of depression was highest among the other ethnic groups (17.6%), followed by Chinese (13.8%), Malays (10.8%) and Indians (6.1%). The results also revealed that depression was more prevalent among the divorcees (42.9%) compared to those who were separated (33.3%), singles (14.0%), widowed (11.5%), and married couples (7.8%). Table 1 shows the association between socio demographic profile and depression.

**Table 1:** Association of socio-demographic profiles with depression among participants.

Profile of the participants	Presence of depression	Absence of depression	Total (n)	Chi square value	df	p-value
	(PHQ-9≥10)	(PHQ-9<10)				
	n (%)	n (%)				
**Gender (n = 1460)**						
Male	66 (12.1)	479 (87.9)	545	3.180	1	0.075
Female	84 (9.2)	831 (90.8)	915			
**Ethnicity (n = 1460)**						
Malay	109 (10.8)	900 (89.2)	1009	9.487	3	*0.023
Chinese	17 (13.8)	106 (86.2)	123			
Indian	18 (6.1)	276 (93.9)	294			
Others	6 (17.6)	28 (82.4)	34			
**Religion (n = 1460)**						
Islam	114 (11.1)	915 (88.9)	1029	10.486	5	0.063
Buddha	11 (13.8)	69 (86.2)	80			
Hindu	13 (5.3)	233 (94.7)	246			
Christian	12 (12.9)	81(87.1)	93			
Others	0 (0.0)	7 (100.0)	7			
None	0 (0.0)	5 (100.0)	5			
**Marital status (n = 1460)**						
Single	71 (14.0)	437 (86.0)	508	23.573	4	**0.001
Married	69 (7.8)	821 (92.2)	890			
Widowed	6 (11.5)	46 (88.5)	52			
Divorced	3 (42.9)	4 (57.1)	7			
Separated	1 (33.3)	2 (66.7)	3			
**Education level (n = 1442)**						
Primary education	18 (14.6)	105 (85.4)	123	2.857	2	0.240
Secondary education	73 (10.3)	639 (89.7)	712			
Tertiary education	58 (9.6)	549 (90.4)	607			
**Employment status (n = 1436)**						
Employed	65 (8.8)	675 (91.2)	740	2.093	2	0.351
Unemployed	70 (11.1)	562 (88.9)	632			
Pension	7 (10.9)	57 (89.1)	64			

*significant at p<0.05 **significant at p<0.001

The detailed results of each factor associated with depression are shown in Table 2 (chronic diseases and history of mental health disorders), Table 3 (anxiety), Table 4 (stressful life events), Table 5 (perceived stress), Table 6 (domestic violence) and Table 7 (self-esteem).

**Table 2:** Association of chronic diseases and history of mental health problems with depression among participants (n = 1460).

Medical history	Presence of depression	Absence of depression	OR (95% CI)	p-value
	(PHQ-9≥10)	(PHQ-9<10)		
	n (%)	n (%)		
**Presence of chronic diseases**				
Yes	56 (15.6)	302 (84.4)	1.83 (1.345–2.50)	**0.001
No	94 (8.5)	1008 (91.5)		
**#Heart disease**				
Yes	5 (16.7)	25 (83.3)	1.64 (0.73–3.71)	0.225
No	145 (10.1)	1285 (89.9)		
**Diabetes**				
Yes	11 (9.6)	103 (90.4)	0.93 (0.52–1.67)	0.819
No	139 (10.3)	1207 (89.7)		
**#Stroke**				
Yes	4 (40.0)	6 (60.0)	3.97 (1.83–8.62)	*0.014
No	146 (10.1)	1304 (89.9)		
**Hypertension**				
Yes	18 (10.6)	152 (89.4)	1.04 (0.65–1.65)	0.886
No	132 (10.2)	1158 (89.8)		
**#Arthritis**				
Yes	6 (16.2)	31 (83.8)	1.60 (0.76–3.39)	0.264
No	144 (10.1)	1279 (89.9)		
**#Cancer**				
Yes	3 (50.0)	3 (50.0)	4.95 (2.19–11.17)	*0.017
No	147 (10.1)	1307 (89.9)		
**Asthma**				
Yes	11 (13.3)	72 (86.7)	1.31 (0.74–2.33)	0.357
No	139 (10.1)	1238 (89.9)		
**#Kidney failure**				
Yes	0 (0.0)	2 (100.0)	1.12 (1.10–1.13)	1.000
No	150 (10.3)	1308 (89.7)		
**#Thyroidism**				
Yes	2 (22.2)	7 (77.8)	2.18 (0.64–7.47)	0.234
No	148 (10.2)	1303 (89.8)		
**#History of mental health problems**				
Yes	16 (53.3)	14 (46.7)	5.69 (3.93–8.25)	**0.001
No	134 (9.4)	1296 (90.6)		
**#Family history of mental health problems**				
Yes	15 (34.1)	29 (65.9)	3.58 (2.30–5.56)	**0.001
No	135 (9.5)	1281(90.5)		
**#Disability**				
Yes	9 (60.0)	6 (40.0)	6.15 (3.95–9.57)	**0.001
No	141 (9.8)	1304 (90.2)		

*significant at p<0.05 **significant at p<0.001

**Table 3:** Association of anxiety with depression among participants (n = 1454).

	Presence of depression	Absence of depression	Total	OR (95% CI)	p-value
	(PHQ-9≥10)	(PHQ-9<10)			
	n (%)	n (%)			
**Anxiety**					
Yes (GAD-7≥8)	80 (67.2)	39 (32.8)	119	12.82 (9.88–16.63)	**0.000
No (GAD-7<8)	70 (5.2)	1265 (94.8)	1335		

**significant at p<0.001

**Table 4:** Association of stressful life events with depression among participants (n = 1458).

	Presence of depression	Absence of depression	Total	OR (95% CI)	p-value
	(PHQ-9≥10)	(PHQ-9<10)			
	n (%)	n (%)			
**Attacked**					
Yes	31 (20.7)	119 (79.3)	150	2.27 (1.59–3.25)	**0.001
No	119 (9.1)	1189 (90.9)	1308		
**Serious illness**					
Yes	17 (14.3)	102 (85.7)	119	1.44 (0.90–2.30)	0.134
No	133 (9.9)	1206 (90.1)	1339		
**#Abused during childhood**					
Yes	11 (31.4)	24 (68.6)	35	3.22 (1.92–5.38)	**0.001
No	139 (9.8)	1284 (90.2)	1423		
**Serious injury due to accident**					
Yes	27 (16.1)	141 (83.9)	168	1.69 (1.15–2.48)	*0.009
No	123 (9.5)	1167 (90.5)	1290		
**Being an orphan before age 10**					
Yes	16 (13.4)	103 (86.6)	119	1.34 (0.83– 2.18)	0.237
No	134 (10.0)	1205 (90.0)	1339		
**Loss of someone very close**					
Yes	29 (9.0)	292 (91.0)	321	0.85 (0.58–1.25)	0.402
No	121 (10.6)	1016 (89.4)	1137		
**#Serious marital problem (n = 890)**					
Yes	5 (13.2)	33 (86.8)	38	1.75 (0.75–4.10)	0.208
No	64 (7.5)	788 (92.5)	852		
**Serious family problem**					
Yes	22 (31.9)	47 (68.1)	69	3.46 (2.36–5.07)	**0.001
No	128 (9.2)	1261 (90.8)	1389		
**Serious financial constraint**					
Yes	37 (20.7)	142 (79.3)	179	2.34 (1.67–3.28)	**0.001
No	113 (8.8)	1166 (91.2)	1279		
**Serious housing problems**					
Yes	20 (32.3)	42 (67.7)	62	3.46 (2.33–5.15)	**0.001
No	130 (9.3)	1266 (90.7)	1396		
**Serious problems at work (n = 739)**					
Yes	8 (14.0)	49 (86.0)	57	1.68 (0.84–3.34)	0.146
No	57 (8.4)	625 (91.6)	682		
**Lost job**					
Yes	14 (26.9)	38 (73.1)	52	2.78 (1.73–4.48)	**0.001
No	136 (9.7)	1270 (90.3)	1406		
**#Legal problems**					
Yes	7 (38.9)	11 (61.1)	18	3.92 (2.15–7.13)	**0.001
No	143 (9.9)	1297 (90.1)	1440		
**Relationship with spouse (n = 981)**					
Unhappy	35 (31.8)	75 (68.2)	110	4.62 (3.20–6.67)	**0.001
Happy	60 (6.9)	811 (93.1)	871		
**Relationship with children (n = 872)**					
Unhappy	20 (32.8)	41 (67.2)	61	6.18 (3.90–9.82)	**0.001
Happy	43 (5.3)	768 (94.7)	811		
**Relationship with family**					
Unhappy	52 (38.5)	83 (61.5)	135	5.20 (3.91–6.92)	*0.001
Happy	98 (7.4)	1225 (92.6)	1323		
**Relationship with work (n = 687)**					
Unhappy	15 (23.4)	49 (76.6)	64	3.32 (1.96–5.62)	*0.001
Happy	44 (7.1)	579 (92.9)	623		

*significant at p<0.05 **significant at p<0.001

**Table 5:** Association of perceived stress with depression among participants (n = 1456).

	Presence of depression	Absence of depression	Total	OR (95% CI)	p-value
	(PHQ-9≥10)	(PHQ-9<10)			
	n (%)	n (%)			
**Perceived stress**					
High (≥15.28)	128 (16.2)	622 (83.8)	790	4.91(3.16–7.62)	**0.001
Low (<15.28)	22 (3.3)	644 (96.7)	666		

**significant at p<0.001

**Table 6:** Association of domestic violence with depression among ever married participants (n = 952).

	Presence of depression	Absence of depression	Total	OR (95% CI)	p-value
	(PHQ-9≥10)	(PHQ-9<10)			
	n (%)	n (%)			
**Domestic violence**					
Yes	19 (46.3)	22 (53.7)	41	7.04 (4.67–10.61)	**0.001
No	60 (6.6)	851 (93.4)	911		

**significant at p<0.001

**Table 7:** Association of self-esteem with depression among participants (n = 1455).

	Presence of depression	Absence of depression	Total	OR (95% CI)	p-value
	(PHQ-9≥10)	(PHQ-9<10)			
	n (%)	n (%)			
**Self esteem**					
Low (<30.15)	130 (15.8)	692 (84.2)	822	5.01(3.16–7.92)	**0.001
High (≥30.15)	20 (3.2)	613 (96.8)	633		

**significant at p<0.001

#### Chronic diseases and history of mental health disorders

The presence of chronic diseases, stroke, cancer, disability, personal and family history of mental health disorders were found to be significantly associated with depression (p<0.05).

#### Anxiety

The comorbidity between depression and anxiety in this study was 67.2%. There was a significant association between anxiety and depression (OR = 12.82, 95% CI: 9.88–16.63).

#### Stressful life events

Twelve stressful life events were found to be significantly associated with depression (p<0.05).

#### Perceived stress

About 790 participants (54.3%) were found to have high perceived stress, based on the mean cut-off point of 15.28. One hundred and twenty eight (16.2%) of the participants with high perceived stress were found to have depression. There was a significant association between high perceived stress and depression (OR = 4.91, 95% CI: 3.16–7.62).

#### Domestic violence (DV)

The prevalence of DV in the community was 4.3% (n = 41). There was a 3-fold increase in the prevalence of DV among females (5.5%) as compared to males (1.6%). Domestic violence was found to be significantly associated with depression (OR = 7.04, 95% CI: 4.67–10.61).

#### Self esteem

Based on the mean cut-off point of 30.15, 822 participants (56.5%) were found to have low self-esteem. One-hundred-and-thirty participants (15.8%) with low self-esteem were found to be depressed and there was a significant association between self-esteem and depression (OR = 5.01, 95% CI: 3.16–7.92).

#### Religiosity

Significant differences were also found between depression and organizational religious activity (p = 0.005), non-organization religious activity (p<0.001) and intrinsic religiosity (<0.001).

### Predictors of depression

Based on the multivariate logistic regression analysis, ten items were found to be predictors of depression. The strongest predictors for depression in descending order were presence of anxiety, serious problems at work, unhappy relationship with children, high perceived stress, domestic violence, unhappy relationship with spouse, low self-esteem, unhappy relationship with family, serious financial constraint and presence of chronic diseases (Table 8).

**Table 8:** Predictors of depression based on multivariate logistic regression analysis.

Predictors	B	Wald	p-value	AOR	95% CI
Anxiety	3.051	85.390	0.000	21.13	11.07–40.36
Serious problems at work	1.585	6.066	0.014	4.88	1.38–17.23
Unhappy relationship with children	1.406	9.456	0.002	4.08	1.67–9.99
High perceived stress	1.194	13.121	0.000	3.30	1.73–6.30
Domestic violence	1.024	4.541	0.033	2.79	1.09–7.14
Unhappy relationship with spouse	0.996	6.612	0.010	2.71	1.27–5.79
Low self-esteem	0.959	10.090	0.001	2.61	1.44–4.71
Unhappy relationship with family	0.768	4.198	0.040	2.16	1.03–4.49
Serious financial constraint	0.716	4.456	0.035	2.05	1.05–4.00
Presence of chronic diseases	0.662	4.622	0.032	1.94	1.06–3.54
Constant	−8.002	6.032	0.014	0.000	

Analysis was based on a sample size of 1428 participants.

The variance accounted by the above covariates after controlling for the other confounders best predicts depression in our study. However, to avoid over-adjustment when considering the role of other covariates, another multivariate logistic regression analysis was done in which all covariates were considered apart from anxiety, high stress and low self-esteem (which could be construed as symptoms of depression). Based on this analysis, predictors of depression (in descending order) were found to be presence of domestic violence, unhappy relationship with children, serious marital problem, unhappy relationship with family, unhappy relationship with spouse, financial constraint, non-organizational religious activity and intrinsic religiosity (Table 9).

**Table 9:** Predictors of depression after removing anxiety, stress and self-esteem.

Predictors	B	Wald	p-value	AOR	95% CI
Domestic violence	1.660	17.889	0.000	5.26	2.44–11.34
Unhappy relationship with children	1.477	15.806	0.000	4.38	2.11–9.07
Serious marital problem	1.446	4.190	0.041	4.24	1.06–16.94
Unhappy relationship with family	1.243	17.266	0.000	3.47	1.93–6.23
Unhappy relationship with spouse	0.937	8.239	0.004	2.55	1.35–4.84
Serious financial constraint	0.868	8.921	0.003	2.38	1.35–4.21
DUREL Non-organizational activity	0.284	13.342	0.000	1.33	1.14–1.55
DUREL Intrinsic religiosity	0.127	8.755	0.003	1.14	1.04–1.24
Constant	−15.395	41.195	0.000	0.000	

Analysis was based on a sample size of 1434 participants.

## Discussion

### Summary of main findings

The prevalence of depression among adults in this study was 10.3% (95% CI: 8.74–11.86). There were ten predictors of depression based on the initial logistic regression model, which were anxiety, serious problems at work, unhappy relationship with children, high perceived stress, domestic violence, unhappy relationship with spouse, low self-esteem, unhappy relationship with family, serious financial constraint and presence of chronic diseases. When reanalyzed using all covariates apart from anxiety, high stress and low self-esteem, eight predictors of depression were found. The results showed five similar predictors which were domestic violence, serious financial constraint and unhappy relationship with children, family and spouse. Serious marital problem, non-organizational religious activity and intrinsic religiosity were found to be the additional predictors of depression.

### Comparison with existing literature

#### Prevalence of depression

The prevalence of depression in this study was 10.3% (95% CI: 8.74–11.86). The result of this study is similar to the findings reported by CDC, which showed that 9.1% of the adults had current depression [7]. However, data from the National Health and Nutrition Examination Survey, 2005 to 2008 found a higher prevalence rate of depression of 21.6% among US adults [40]. This survey also used the PHQ-9 to detect depression. The higher prevalence rate was probably due to the lower cut-off point of >5 that was used to classify depression. When the cut-off point of ≥10 was used, the point prevalence of depression decreased to 6.8%.

The 2006 BRFSS that was conducted among adults aged 18 years and above in 41 states and territories in US found a 8.7% prevalence of current depression by using the PHQ-8 [22]. Women, those with less than high school education, those who were previously married and singles were significantly associated with current depression [22]. Depressed adults were 14.9 times more prone to suffer from anxiety than those without depression. The adjusted prevalence of current depression in urban areas in South India which was conducted among 25,455 adults aged 20 years and above from June 2001 to August 2002 was 15.9% [41]. Females, low income group, divorced and widowed were significantly associated with depression.

Findings of this study correspond to a study conducted in the community of Selangor among adult women, which found a prevalence of depression of 8.3% [42]. This study's result also corresponds to another study conducted in the East Coast of Peninsular Malaysia which found that the prevalence of depression was 11.3% among the population in their communities [43]. The study was carried among 520 residents in the rural community of Terengganu, Pahang and Kelantan from April 2009 until January 2011. Slightly higher prevalence rates were found in studies conducted among patients in primary healthcare settings in Kuala Lumpur, the capital city of Malaysia (14.4%) [44], and Selangor (12.1%) [45].

It is noted that studies which used specific instruments to diagnose depression, instead of screening instruments as in this study have lower prevalence rates of depression. For example, results from the National Epidemiologic Survey on Alcoholism and Related Conditions (NESARC) which was conducted in year 2001 to 2002 in the US found that the 12-month prevalence of MDD was 5.28% among 43,093 adults in the community of United States [8]. The Alcohol Use Disorder and Associated Disabilities Interview Schedule–DSM-IV Version (AUDADIS-IV) from the National Institute on Alcohol Abuse and Alcoholism was used to determine MDD. Females, middle aged, widowed, separated, or divorced and low income groups were found to be significantly associated with MDD.

A study conducted by Amoran et al in urban and rural areas of Oyo State of Nigeria among 1,105 adults in the community, found that the overall prevalence of psychiatric morbidity was 21.9%, and the point prevalence of depression was 5.2% [46]. The earlier prevalence rate was determined by using the screening instrument, General Health Questionnaire-12 (GHQ-12); while the latter was determined by using a diagnostic instrument, the Structured Clinical Interview DSM-IV. The prevalence of depression was higher in rural areas compared to urban areas due to lack of basic facilities in rural areas.

In Malaysia, the recent NHMS IV [17], which used the MINI to diagnose depression, found a much lower prevalence of current depression (1.8%) among adults aged 16 years and above. While another study by Chong et al on the prevalence and impact of MDD among Chinese, Malays and Indians in an Asian multi-racial population found a lifetime and twelve months prevalence of MDD of 5.8% and 2.2% respectively [13]. The face-to-face interviews were conducted among 6,616 adults Singapore residents aged 18 years and above using the English, Chinese and Malay versions of World Mental Health Composite International Diagnostic Interview to detect MDD. MDD was found to be significantly higher among the females, Indians and among those who were divorced, separated and widowed.

#### Predictors of depression

The main predictors of depression in this study were presence of anxiety, serious problems at work, unhappy relationship with children, high perceived stress, domestic violence, unhappy relationship with spouse, low self-esteem, unhappy relationship with family, serious financial constraint and presence of chronic diseases. The strongest predictor of depression in this study was anxiety (OR = 21.13, 95% CI: 11.07–40.36). Co-morbid depression and anxiety has been demonstrated extensively locally and globally [22]. Results from the NESARC showed that anxiety disorders were strongly correlated with MDD [8]. In our study, the prevalence of comorbid depression and anxiety was 67.2%.

This study found that the prevalence of depression was higher among adults with chronic diseases compared to those without any chronic disease. Data from the WHO World Health Survey among 245,404 adults aged 18 years and above from 60 countries found that significant decrements in health have been shown to be associated with depression, commonly in chronic diseases, and higher in diabetes [21]. The study by Chong et al among adults in Singapore, found that almost 50% of the adults with MDD presented with at least one type of chronic disease [14]. A review on the vital link between chronic diseases and depressive disorders by Chapman et al [47] found that there was an interrelationship between depressive disorders and chronic diseases, where depressive disorders were seen to precipitate chronic diseases, and the presence of chronic diseases worsen the symptoms of depression. Clarke and Currie who reviewed a total of 159 systematic reviews, meta analyses and evidence-based clinical practice guidelines published between 1995 and 2007 on epidemiology, risk factors and managements concluded that there was a strong association between physical illness and depression [48]. Lim and colleagues analyzed data from the National Mental Health Survey of Adults in Singapore [NHMS (A)] that was conducted among 2,801 Singapore citizens and permanent residents aged 20–59 years from 15 February 2003 to 30 March 2004 [18]. They found that participants with anxiety and depression were associated with chronic diseases, among which 59% of them had at least one type of chronic diseases and were also shown to have lower functional well-being and quality of life.

Domestic violence was one of the significant predictors of depression in this study. The prevalence of domestic violence in this study was 4.3%. This finding is much lower than the findings from the WHO multi-country study on women's health and domestic violence, which showed a lifetime and past twelve-months prevalence of physical or sexual violence, or both of 15% to 71% and 4% to 54%, respectively [49]. The WHO study was conducted among 24,097 women in ten countries between the years 2000 to 2003. The investigators commented on the wide range of prevalence in domestic violence and will further explore the differences in future. A study by Fikree et al on the domestic violence and health among one-hundred and fifty Pakistani women in outpatient clinics found that the lifetime prevalence of physical violence was 34%. Almost 73% of the women who were physically abused were found to be depressed. Severity of physical abuse was found to be the significant predictor of depression [50]. Strong significant association was observed between depression and ever been afraid of a partner [51]. Women who were abused had greater risk of psychiatric morbidity as compared to men [52]. It is understood that women who were afraid of their partners were 36 times prone to be abused than those who were not afraid. Besides that, abused women were also found to have high depression scores [53]. In a cohort study on intimate partner violence (IPV) and depression from 1994–1997 among 548 couples in rural areas of Iowa by Renner et al showed that physical IPV was found among 11.5% and 8.2% of males and females, respectively [54]. Higher levels of depressive symptoms and strong correlation with abuse were found among females as compared to males.

The association between depression and stressful life events has been demonstrated extensively. This study found that depression was significantly associated with stressful life events such as serious problems at work, unhappy relationship with children, spouse and family, as well as serious financial constraint. Other studies also found that depression was significantly associated with serious financial constraint [55], problem at work [56] and unhappy relationship with spouse, children and family [45]. Depressed persons were also observed to be at high risk to experience subsequent stressful life events [57].

In a case control study that was conducted between April 2010 to December 2010 among 188 young adults, aged 20–35 years in a primary care setting in Mallorca, Spain also found that depression was significantly associated with being separated, divorced and widowed, having problems at work, problems in relationships, and maintaining close relationships for almost most of the time [58].

Lee who analyzed the data from the First Korean Working Conditions Survey conducted in 2006 among 7,071 employees aged between 15 to 64 found that 6.3% and 5.3% of the population had moderate and high job insecurity, respectively [23]. The study found that women and temporary employees had higher sense of job insecurity compared to the other groups. Depression was found to be associated with temporary employees with higher job insecurity; whereas among permanent employees, depression was associated with moderate level of job insecurity. A study among 315 health care professionals in a critical care and emergency unit in Spain found that depression was associated with violence at work, mainly physical aggression [59]. Oliver-Quetglas and colleagues found that the control group (those without depression) were found to have higher income and job stability compared to those with depression [58].

Another predictor for depression found in this study was financial constraint. Economic hardship was also found to be associated with increased level of depression by the Fragile Families and Child Well-being Study (FFCWS), a longitudinal study that was conducted from 1998 to 2000 [60]. This study conducted among 1304 mothers and 1230 fathers revealed that economic hardship was found to be associated with higher relationship distress and increased levels of depression for both the mothers and fathers. The presence of depression was also found to lead to higher relationship distress.

Perceived stress was also found to be a significant predictor of depression in this study. As found in a study by Sangon among 142 women aged 18 years and above in Thailand, increased perceived stress was shown to have greatest effect on severity of depression [61]. Another study by Yaacob et al on relationship between loneliness, stress, self-esteem and depression among 1,407 secondary school adolescents, aged between 13 to 17 years old in Malaysia, found that participants with higher stress scores had higher depression scores [62].

Self-esteem was another predictor of depression in this study. Sowislo and Orth who reviewed 80 articles from years 1984 to 2010 in their meta-analysis of longitudinal studies confirmed that low esteem contributes to depression and is not influenced by age, gender, instruments used to measure self-esteem and the time lag between the assessments [63]. These findings are supported by Orth et al who compared data from three longitudinal studies and also found that low self-esteem and stressful life events are independent risk factors for depression [57].

Serious marital problem was found to be an additional predictor for depression in this study. Maciejewski et al explored the sex differences in event related risk for depression among 1024 men and 1800 women [64], and found that both men and women with marital problems were at risk for depression. In another study, Kendler et al found that serious marital problems elevated the risk for the onset of depression [65].

Another significant predictor for depression in this study was religiosity. When reanalyzed (after excluding anxiety, high stress and low self-esteem), two major dimensions of religiosity which were non-organizational religious activity and intrinsic religiosity were found to also be the predictors of depression. This was a surprising finding as two studies and a recent systematic evidence-based review showed protective effects of religiosity on depression [66]–[68]. Another study in Malaysia found that religiosity also had a protective effect on distress [69]. However, our findings showed that participants with higher scores for religiosity were at higher risk of developing depression. A study by Azorin et al on religious involvement in major depression among 493DSM-IV major depressive patients had similar findings to our study [70]. They found that patients with high religious involvement had higher scores of depressed mood compared to those with low religious involvement. It was also observed that religious involvement has negative effect in depressive patients, where it was found that high religious involvement correlated with depressive temperament and chronic depression.

A study conducted in Gaza Strip among adolescents on impact of war and religiosity on psychiatric disorders also found that religiosity predicted depression [71]. According to this study by Khamis V, adolescents with high religiosity level demonstrated higher levels of depression and anxiety. In another study which was conducted among university students, Maltby et al who explored the relationship between religiosity and depression found that higher depression scores in men were accounted by extrinsic-personal and social religious orientation, while higher depression scores in women were associated by extrinsic social religious orientation [72]. Based on our findings, we suggest the possible explanation for high religiosity and depression could be that the depressed participants sought religion as a means of coping with their problems, and used religion to find relief and solace. This finding has brought up a new aspect which should be explored further in future community studies in Malaysia.

### Strengths

The main strength of this study is that the sampling was conducted by the DOS, to limit the biasness and improve the accuracy in selecting the participants. Besides that, the use of the validated Malay version of the PHQ-9 facilitates the detection of depression in this study as most of the participants were more fluent and comfortable in their national language.

### Limitations

The limitation of this study is that the data were self-reported by the participants which may incur some recall and reporting bias. However, the participants were requested to think carefully and answer as honestly as possible. Another limitation of this study is such that it is a cross-sectional study design which limits the determination of the temporal relationship between the studied independent variables and depression to establish the cause-effect relationship.

## Conclusion

The prevalence of depression in this study is similar to that found in other studies. The predictors of depression in the community of Selangor have been identified and the findings from this study can serve as a baseline data to develop effective community programs to assist in the management of common mental health disorders, mainly depression.

## Acknowledgments

We would like to thank the Department of Statistics Malaysia (DOS), Faculty of Medicine and Health Sciences, University Putra Malaysia (UPM) and all the participants who were involved in this study.
